# Correlating Ratings of Health Insurance Plans to Their Providers' Attributes

**DOI:** 10.2196/jmir.6475

**Published:** 2016-10-24

**Authors:** Prajna Shetty, Ryan Rivas, Vagelis Hristidis

**Affiliations:** ^1^ University of California, Riverside Riverside, CA United States

**Keywords:** health insurance, doctor reviews, doctor attributes, insurance plans quality

## Abstract

**Background:**

There is a push towards quality measures in health care. As a consequence, the National Committee for Quality Assurance (NCQA) has been publishing insurance plan quality measures.

**Objective:**

The objective of this study was to examine the relationship between insurance plan quality measures and the participating providers (doctors).

**Methods:**

We collected and analyzed provider and insurance plan data from several online sources, including provider directories, provider referrals and awards, patient reviewing sites, and hospital rankings. The relationships between the provider attributes and the insurance plan quality measures were examined.

**Results:**

Our analysis yielded several findings: (1) there is a moderate Pearson correlation (*r*=.376) between consumer satisfaction insurance plan scores and review ratings of the member providers, (2) referral frequency and provider awards are negligibly correlated to consumer satisfaction plan scores (correlations of *r*=.031 and *r*=.183, respectively), (3) there is weak positive correlation (*r*=.266) between the cost charged for the same procedures and consumer satisfaction plan scores, and (4) there is no significant correlation between member specialists’ review ratings and specialty-specific insurance plan treatment scores for most specialties, except a surprising weak negative correlation for diabetes treatment (*r*=-.259).

**Conclusions:**

Our findings may be used by consumers to make informed choices about their insurance plans or by insurances to understand the relationship between patients’ satisfaction and their network of providers.

## Introduction

There are several health insurance marketplaces and search portals (eg, ehealthinsurance.com) that help individuals and small employers shop for, select, and enroll in high-quality, affordable health plans. Insurance plans are generally ranked based on relative quality and price. These marketplaces and search portals need to establish criteria and selection processes for quality measures. Most of them measure the quality of health plans by surveying plan enrollees on their satisfaction with their coverage and then publishing quality and satisfaction data online [1]. However, the relationship between the quality of insurance plans and the properties of providers in their networks has not been adequately studied, which is the focus of this study.

We collected a rich set of data for each provider ranging from average patient review scores, referral patterns, affiliated hospital scores, relative costs, and provider awards. Specifically, we used data collected from Centers for Medicare & Medicaid Services (CMS) and provider profile websites on a set of 600,000 US health care providers. We also collected ranking data from other sources; specifically, U.S. News was used for specialty-specific hospital rankings. We converted each provider’s information to a set of intuitive qualitative attributes. For instance, affiliated hospitals were mapped to specialty-specific rankings to assign a score to the affiliated hospitals of a provider relevant to their specialty. As a peer-nominated award, we selected the Castle Connolly award. Each year, Castle Connolly distinguishes top providers both nationally and regionally through a peer nomination process that involves over 50,000 providers, and hospitals and health care executives [2]. Similarly, we collected quality data from National Committee for Quality Assurance (NCQA) for each insurance plan ranging from state, plan category, ranking, overall review scores, customer satisfaction scores, as well as preventive care and treatment scores [3].

We then adopted a data-driven approach to determine if the provider attributes were correlated with the insurance quality indicators. Specifically, we measured the correlation between several provider attributes (reviews rating, awards, affiliated hospitals, etc) of member providers of an insurance plan to key quality scores of the insurance plans.

Key challenges to our data collection and analysis included mapping providers from CMS to providers in provider profile sites, mapping insurance names between accepted insurances obtained from provider profile sites and insurances obtained from NCQA, and mapping hospital names between each source. These challenges are due to the lack of a common identifier for providers, insurance plans, or hospitals across the data sources.

There have been several studies to determine the quality of health insurance plans. These studies can be split into two categories: (1) health insurance marketplaces and search sites, and (2) attributes associated with health plan quality.

### Online Health Insurance Marketplaces and Search Sites

There are several health insurance marketplaces, authorized by the Affordable Care Act, that help individuals and small employers shop for, select, and enroll in high-quality, affordable private health plans. In fact, the Affordable Care Act requires the US Department of Health & Human Services to develop quality data collection and reporting tools such as a quality rating system, a quality improvement strategy, and an enrollee satisfaction survey system [1]. Information from the quality rating system, quality improvement strategy, and surveys will inform consumer selection of a quality health plan, decisions about quality health plan certification, and the Federal and State marketplaces’ monitoring of quality health plan performance. All these measures use data collected through consumer experience surveys such as enrollee experience surveys and health insurance marketplace surveys. Other insurance search sites, such as einsurance.com and insure.com, collect user feedback regarding each interaction with their partner insurance providers. This feedback enables them to identify potential customer service issues and is also used as an essential component of the ranking system that they use to determine how these partners are presented to prospective future clients [4,5]. Hence, most of these studies focus on user-generated content and do not consider the rich set of provider data readily available. Research is lacking on the association between information from providers in the network with the respective health insurance plans. For example, if patients rate insurance plans based on cost, are these ratings useful for finding providers that provide quality health care?

### Attributes Associated With Insurance Quality

Several surveys have examined the quality of health insurance plans based on consumer feedback and have tried to determine attributes associated with insurance quality. Feldman states that a cornerstone of high-quality integrated care for people with medical, behavioral, and long-term services and support needs is a dynamic person- or family-centered plan of care built on significant individual and caregiver involvement and comprehensive assessments and reassessments over time to capture changes in people’s circumstances and preferences. Other key ingredients identified were (1) a multidisciplinary care team with one accountable care coordinator, and (2) a comprehensive provider network with a strong primary care base and a range of other providers and services that can accommodate diverse needs throughout a lifetime [6].

URAC (Utilization Review Accreditation Commission), which is an independent, nonprofit organization known for promoting health care quality through its accreditation, education, and measurement programs, addresses the following key areas aimed at helping plans deliver safe, high-quality, patient-centered, high-value care: Wellness and Health Promotion; Care Coordination; Medication Safety and Care Compliance; Rewarding Quality; Care Delivery through a Network; Mental Health Parity; Measures—patient centeredness, coordination of care, patient safety, health plan administration, efficiency, effectiveness of care and health information technology integration; and Patient Experience of Care (Consumer Assessment of Healthcare Providers and Systems Survey) [7]. In our study, we examine the correlation of provider attributes to quality indicators of health insurance plans.

## Methods

### Summary

**Figure 1:**
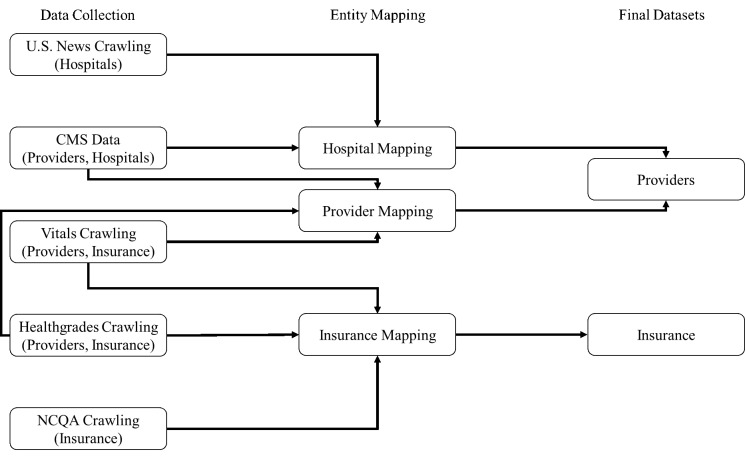
Visual description of data preprocessing.

For the purpose of our data-driven analysis, we have collected a large amount of information about US health providers, mainly physicians, from multiple online sources including the CMS data on providers and hospitals, U.S. News rankings of hospitals, and additional provider information and reviews from provider profile websites. We have also collected information about the rankings of private, Medicare, and Medicaid health insurance plans from NCQA. We then mapped entities across sources to create a database of providers and health plans. Figure 1 shows the process of mapping insurances accepted by the providers and the insurance plans obtained from NCQA. We then used this providers’ information and insurance information database in each of our analyses.

### Data Collection

Insurance information and patient ratings of providers were collected from both Vitals and Healthgrades [8,9]. Hospital rankings were collected from U.S. News reports [10,11]. Additionally, insurance plan rankings for 2014-2015 were collected from NCQA. We also used the datasets released by CMS for health care providers (and hospitals) based in the United States. This information includes general information such as the provider’s specialties, medical training, and hospital affiliations [12,13]. Other provider information includes the Healthcare Common Procedure Coding System (HCPCS), physician referrals, and prescription data [14-16]. Note that all CMS datasets link providers using a National Provider Identifier (NPI). CMS hospital information includes names, location, and a unique identifier, which is used to link each NPI to their affiliated hospitals. CMS data were downloaded directly from CMS websites. Separate crawlers were built using jsoup [17], a Java library for obtaining and parsing webpages, for each of the other data sources: Vitals, Healthgrades, U.S. News, and NCQA.

Aggregating the datasets posed unique challenges for entity mapping, such as mapping providers from Healthgrades to providers in CMS, as described in the next section. In total, we collected information on 3.2 million distinct providers from CMS, 4600 distinct hospitals from CMS, 1.9 million distinct providers from Healthgrades, one million distinct providers from Vitals, and 1956 hospitals from U.S. News. We also collected information of 1264 health plans from NCQA. Of these, NCQA has ranked 1051 plans based on clinical performance, member satisfaction, and results from NCQA Accreditation surveys. The remaining insurances had partial data. After appropriate data transformations and entity mappings, we generated the set of provider attributes listed in Table 1 and health insurance plan attributes listed in Table 2.

**Table 1:** List of provider attributes used in our analysis based on the data collected.

Category	Attribute	Description	Source	Min.	Max.	Mean	Median
General information	NPI	National Provider Identifier.	CMS	N/A	N/A	N/A	N/A
	Gender	Male or Female, as specified in the CMS data.	CMS	N/A	N/A	N/A	N/A
	Specialties	A set of attributes, one for each specialty, eg, cardiologist.	CMS	N/A	N/A	N/A	N/A
From peers	NumReferrals	Normalized number of referrals.	CMS	0	4018	70.1	10
	Castle Connolly	Whether or not the provider is recognized by Castle Connolly as a distinguished provider.	Vitals	N/A	N/A	N/A	N/A
Average rating from patient reviews	UserRatings	Overall review score assigned by user (patient).	Reviews from Vitals and Healthgrades	0	100	82.06	87.5
	NumReviews	Number of patient reviews for the provider.	N/A	0	247	0.96	0
Insurance	NumInsurances	Number of insurers accepted by the provider.	Vitals and Healthgrades	1	8	1.7	1
	IndividualInsurers	A set of attributes, one for each insurer accepted by the provider, eg, Humana.	Vitals and Healthgrades	N/A	N/A	N/A	N/A
Hospital affiliations	HospitalRanking	The ranking of the provider’s affiliated hospitals.	CMS (hospitals) and U.S. News (ranks of hospitals)	N/A	N/A	N/A	N/A

**Table 2:** List of health insurance attributes used in our analysis based on the data allocated. All attributes in this table are from NCQA.

Category	Attribute	Description
General information	PlanName	Insurance plan name.
	State	The state to which the plan belongs.
	PlanCategory	The category of the plan, eg, private, Medicare, Medicaid.
	PlanType	The type of the plan, eg, preferred provider organization (PPO), health maintenance organization (HMO).
Quality indicators – Overall	Rank	The overall rank of the plan.
	OverallScore	The overall score of the plan.
Quality indicators – Customer service	OverallConsumerSatisfactionScore	The score for consumer satisfaction.
	GettingCareScore	Scores based on appointments, preventive care, test, and easy and quick access to treatments.
	SatisfactionWithPhysiciansScore	Scores based on providers, care revived and health promotion and education.
	SatisfactionWithHealthPlanServicesScore	Scores based on handling claims and other plans services.
Quality indicators – Prevention	OverallPreventionScore	The score for preventive care.
	ChildrenAndAdolescentsScore	Scores based on well-child visits, immunizations, nutrition counseling, physical activity counseling.
	Women’sReproductiveHealthScore	Scores based on prenatal checkup and postpartum care.
	CancerScreeningScore	Scores based on various cancer screenings.
	OtherPreventiveServicesScore	Scores based on flu vaccinations, chlamydia screening, and other preventive care.
Quality indicators – Treatment	OverallTreatmentScore	The score for different treatments.
	AsthmaTreatmentScore	Scores based on asthma medication and treatment.
	DiabetesTreatmentScore	Scores based on blood pressure control, glucose testing and control, low-density lipoprotein cholesterol screening and control, monitoring kidney diseases.
	HeartDiseaseTreatmentScore	Scores based on controlling blood pressure and cholesterol and beta-blockers after heart attack.
	MentalAndBehavioralHealthScore	Scores based on depression medication, alcohol and drug dependence treatment, etc.
	OtherTreatmentMeasuresScore	Scores based on monitoring key long-term medications, antibiotic use, testing for chronic obstructive pulmonary disease, etc.

### Entity Mappings

The names of insurance obtained from Vitals and Healthgrades differ from the names of insurance in the NCQA data. For example, “United Healthcare Services, CA” and “United Healthcare, CA” refer to the same insurance plan, as do “Aetna Life Insurance, AR” and “Aetna HMO, AR”. In order to achieve this mapping, we used the Levenshtein distance metric [18] to map Healthgrades and Vitals insurance to NCQA insurance. This generated 242 mappings between Vitals and NCQA insurance and 1330 mappings between Healthgrades and NCQA insurance.

The hospital rankings listed by U.S. News categorize hospitals across several specialties for adults and children; for each hospital listed, the hospital’s score, name, and location were collected for each specialty for both adults and children. Further, the hospital specialties reported by U.S. News do not always correspond to the specialties listed by CMS. In particular, CMS uses a taxonomy of medical specialties that consider subspecialties, whereas U.S. News uses broad categories of specialties [19]. Note that this mapping is not necessarily one-to-one; for example, a provider specializing in internal medicine may map to several categories listed by U.S. News. Therefore, we manually mapped all specialties with more than 100 occurrences to the specialties used by U.S. News. This generated 5651 mappings. We then used these mappings to assign scores to each of the affiliated hospitals, using the average for a hospital’s score when the provider’s specialty mapped to more than one specialty listed by U.S. News. We then assigned HospitalScore to the hospital affiliation with the maximum score, where null values are used for providers whose hospital affiliations are missing from the mappings. Also, for each HCPCS code of a provider, we computed the amount charged for this provider, relative to others of same specialty in the area (1000 closest within a 30-mile radius, normalized to a range of 0 to 100, where 100 goes to the most expensive physician). We then took the weighted average (by the number of procedures of a provider) of these relative charges to get the relative cost with respect to area.

In order to identify Castle Connolly and patient reviews information for each provider, CMS providers needed to be mapped to Vitals and Healthgrades provider profiles. This mapping exercise allowed us to map 608,935 providers between CMS, Vitals, and Healthgrades, 25,514 of whom have received a Castle Connolly award. To map CMS providers to providers in the other sources (Heathgrades and Vitals), we followed a hybrid automatic-manual data integration approach. First, we identified a promising set of attributes to use for mapping, specifically, first name, middle name, last name, address, medical school, graduation year, affiliated hospitals, and specialties. For each attribute, we constructed a customized mapping algorithm. For example, the mapping between first names is computed using the Levenshtein distance between the two strings. Then, we assigned weights to each attribute matching score based on a large number of accuracy experiments, where the authors defined the ground truth mappings. We then computed a mapping threshold based on the mapping scores via more accuracy experiments. Note that each Vitals/Healthgrades provider is mapped to at most one CMS provider, so no duplicate provider data are present in the final dataset.

Only 4% of all mapped providers have received a Castle Connolly award, and 42% of all mapped providers have zero referrals. A majority of providers with zero referrals specialized in Internal Medicine, Family Medicine, or Emergency Medicine. Also, 213 of 1264 health plans collected had incomplete data. In order to correlate rank of affiliated hospitals and insurance scores, we needed the rank of the hospitals. However, only 50 out of the 1956 hospitals obtained from U.S. News were ranked. We considered the unranked hospitals to be at the bottom of the list. We then took the median of the unranked hospitals (ie, 1053) and considered this to be the rank of the unranked hospitals. Also, in order to account for local trends, we performed our analysis at both the national and state levels. Health care is regulated at both the state and federal levels. These regulations, along with demographics and population health, create localized trends in health care.

## Results

### Summary

The results of our analysis consist of a description of general statistics about the different types of insurance and a state-wise analysis of the consumer satisfaction insurance plans. Then we report on correlations between insurances’ consumer satisfaction score and the average patient review scores of providers that accept those insurances. We report similar correlations between insurances’ overall NCQA consumer satisfaction score and then average number of referrals per provider, ratio of Castle Connolly providers, average affiliated hospital scores of providers, and relative cost of providers with respect to area. Last, we break down the providers according to their specialties and describe correlations between the average patient review scores and treatment insurance scores for condition-specialty combinations.

### General Statistics of Insurance Plans

We first analyzed general statistics about the various insurance plans at the national level. We calculated the average overall consumer satisfaction scores of the insurance plans (see corresponding row in Table 2), where we average across the types of insurance plans: private, Medicare, and Medicaid. We also calculated the average patient review scores of providers (referred as “UserRatings” in Table 1) accepting these different types of insurances. Our findings are shown in Table 3 along with the statistical analysis. The patient review scores are on average higher than the insurance satisfaction scores, and with high significance for private PPOs and Medicare plans.

**Table 3:** General statistics about different types of health insurance plans.

Insurance plan type	Average patient review score (*P* value)	Average consumer satisfaction insurance score (*P* value)
Private PPO	82.03 (<.001)	79.75 (.384)
Private HMO	82.54 (<.001)	81.63 (<.001)
Medicaid	82.78 (<.001)	77.52 (<.001)
Medicare PPO	82.39 (<.001)	76.71 (.263)
Medicare HMO	81.55 (<.001)	76.9 (.123)

**Figure 2:**
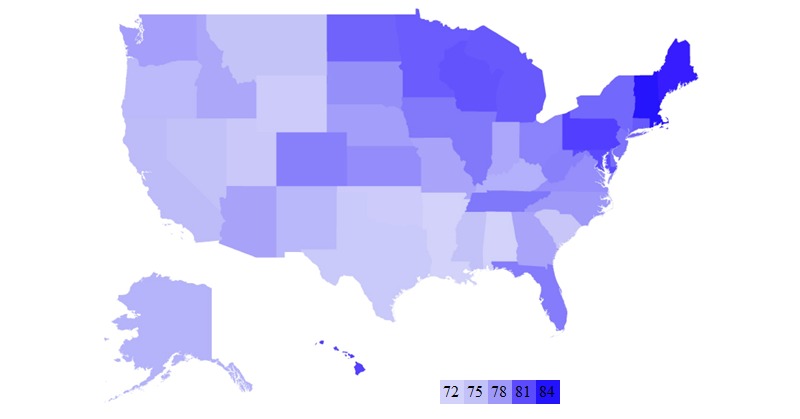
Heat map showing average consumer satisfaction insurance scores of different plans.

**Figure 3:**
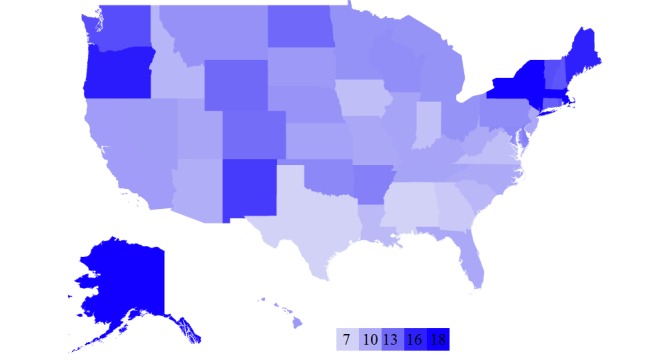
Heat map showing number of health care providers per 1000 people in each state.

**Figure 4:**
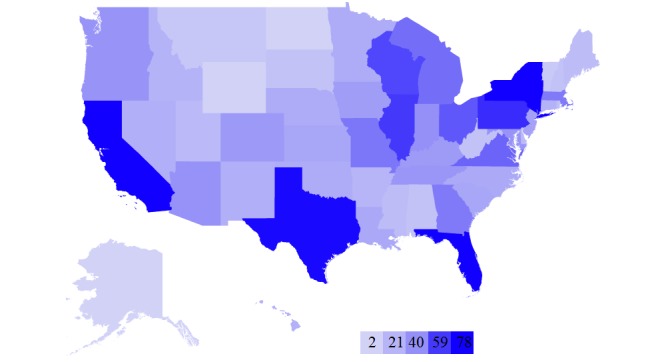
Heat map showing the number of health insurance plans evaluated by NCQA per state.

To estimate significance between values in the same row of Table 3, the Wilcoxon signed-rank test significance values are as follows, between average patient and insurance scores: private PPO˂.001, private HMO=.13, Medicaid=.008, Medicare PPO˂.001, and Medicare HMO˂.001. To compute significance of a value with respect to the union of the other four plan types in the same column (*P* value), we used the Mann-Whitney U test.

We also computed the average consumer satisfaction insurance scores for each state. The heat map in Figure 2 shows our findings. The darker colored states are those that have a higher overall consumer satisfaction insurance score while the lighter ones have lower consumer satisfaction insurance scores. From the map, we can conclude that northeastern states have higher consumer satisfaction insurance scores.

Similarly, we computed the number of health care providers per 1000 people for each state. As shown in Figure 3, the darker colored states have more providers per capita while the lighter states have fewer per capita. From this map, we can see that the northeastern states also tend to have more health care providers per capita.

Finally, we counted the number of insurance plans evaluated by NCQA per state. The heat map in Figure 4 shows our results. The darker colored states have more insurance plans while the lighter ones have fewer. The map shows that the most populous states have the most insurance plan options while the less populous states tend to have fewer.

### Attribute Correlations

**Figure 5:**
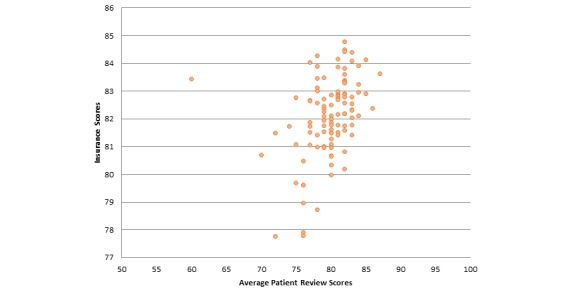
Correlation between average patient review scores and consumer satisfaction insurance scores (overall) (correlation coefficient=.376, *P* ˂.001).

We computed the Pearson correlation of average patient review scores of providers that accept a particular insurance plan and that insurance plan’s NCQA scores. We found that there is a moderate positive correlation between these attributes (specifically .376). Figure 5 illustrates this correlation. We then did the same analysis state-wise and found that the Pearson coefficient increases in value, showing greater correlation when we localize the analysis. Table 4 shows the correlation coefficient between these same attributes for some of the different states. A couple of interesting observations can be made based on these correlations. First, there seems to be a moderate correlation between average patient review scores and consumer satisfaction insurance scores. Hence, insurance that includes providers with good reviews is more likely to have a better overall score. Also, the correlation between these two attributes seems to get stronger when we break down the data state-wise.

**Table 4:** Correlation between average patient review scores and consumer satisfaction insurance scores.

State		Correlation
Overall		.376
**State-wise**		
	New York	.869
	Texas	.794
	Illinois	.738
	Pennsylvania	.696
	California	.647
	Ohio	.549
	Florida	.457

**Figure 6:**
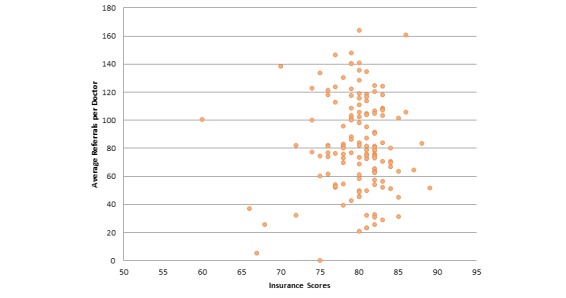
Correlation between average referrals per provider and consumer satisfaction insurance scores (correlation coefficient=.031, *P*=.715).

**Figure 7:**
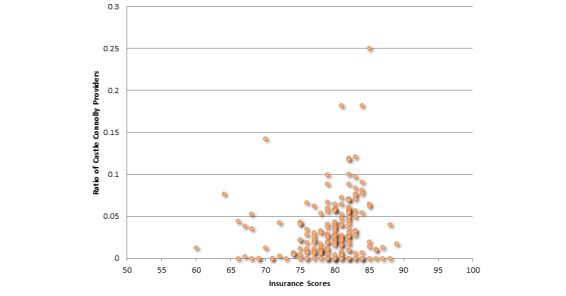
Correlation between ratio of Castle Connolly providers and consumer satisfaction insurance scores (correlation coefficient=.183, *P*=.001).

**Figure 8:**
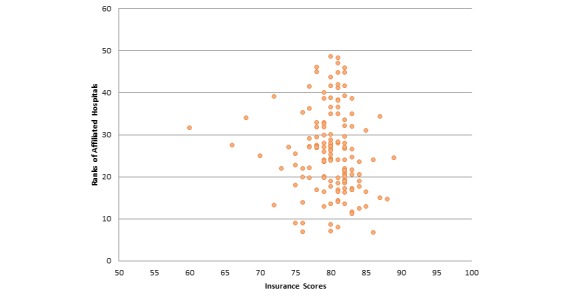
Correlation between ranks of affiliated hospitals and consumer satisfaction insurance scores (correlation coefficient=.108, *P*=.199).

**Figure 9:**
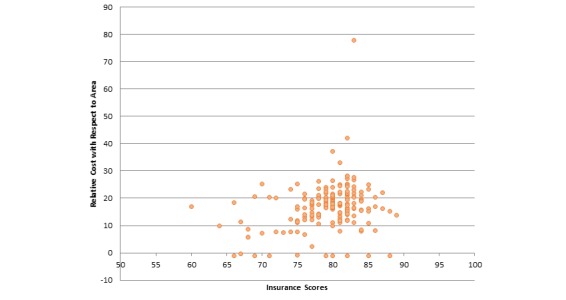
Correlation between relative cost of providers with respect to area and consumer satisfaction insurance scores (correlation coefficient=.266, *P*<.001).

Next, we report correlations between average referrals per provider for insurances and those insurances’ NCQA scores. Our analysis showed that there is a positive but very low correlation (specifically .031) between these two attributes. Hence, referral frequency of providers is negligibly correlated to consumer satisfaction insurance scores. Figure 6 further illustrates this correlation. Figure 7 illustrates the correlation between ratios of providers having the Castle Connolly award to the overall insurances’ NCQA scores. We found a positive but negligible relationship between these attributes, specifically .183. Hence, whether a provider has received a Castle Connolly award or not does not affect the insurances’ overall score. With respect to correlation between average ranks of affiliated hospitals and consumer satisfaction insurance scores, there exists a negative but negligible correlation between these two attributes (specifically -.108). Since we are considering ranks of hospitals, the negative correlation is expected. Hence, consumer satisfaction insurance scores are unlikely to be affected by the ranks of affiliated hospitals of the providers under that insurance plan. Figure 8 illustrates this correlation. We also determined the correlation relationship between relative cost of providers with respect to area and the consumer satisfaction insurance scores. Our findings showed a weak positive correlation of .266 between these two attributes. Figure 9 shows this correlation.

We then examined correlations between average patient review scores for specialist providers and the NCQA treatment insurance scores for these specialties. For this we used the individual treatment scores obtained from NCQA for the various conditions described in Table 2. We then compared these scores to the average patient review scores of only those providers that provide that kind of care, as shown by the mapping of condition to specialties in Table 5. For example, the average patient review scores of pediatricians were compared to the NCQA scores for treatment of children and adolescents. Table 5 lists our findings. We observed that for women’s health, mental and behavioral health, and cancer screening there exists a positive but negligible correlation between the average NCQA scores and the average patient review scores. However, for heart diseases, child and adolescent health, and diabetes, there exists a negative and negligible to weak correlation between the attributes.

**Table 5:** Conditions and associated specialties ranked by correlation between NCQA scores and average patient review scores.

Condition from NCQA	Corresponding member specialties	Correlation of treatment insurance score with average patient review score
Women’s health	Obstetrics and Gynecology, Gynecology Oncology	.135
Mental and behavioral health	Counselor, Psychoanalyst, Clinical Neuropsychologist, Psychologist, Psychoanalysis, Marriage and Family Therapist	.112
Cancer screening	Pediatric Oncology, Oncology, Hematology & Oncology, Radiation Oncology	.112
Heart disease	Cardiologist, Cardiac Rehabilitation, Cardiology Technician, Cardiovascular Diseases	-.002
Children and adolescent health	Pediatrics, Neonatal Pediatrics, Pediatrics Critical Care	-.083
Diabetes	Diabetes Educator, Endocrinology, Diabetes and Metabolism	-.259

## Discussion

### Principal Findings

Our analysis shows that there are several provider attributes that are correlated to insurance quality attributes. We showed that patient review scores for providers are correlated to consumer satisfaction insurance scores. This is expected given that patients who are happy with the care they receive from their providers are more likely to also be happy with their overall insurance plan. For example, if a patient has complaints about the billing at a provider’s office, this patient will likely be unhappy with the insurance company who did not help cover or settle the bill.

On the other hand, our results showed negligible correlation between average referrals per provider and consumer satisfaction insurance scores. This is not surprising, as there is no convincing evidence that a higher number of referrals is connected to better skills for a provider or to better relationship with patients. Similarly, we demonstrated that there is a negligible correlation between the ratio of Castle Connolly providers and the consumer satisfaction insurance scores.

The case between rank of affiliated hospitals and consumer satisfaction insurance scores was similar. However, we found a weak positive correlation between the relative cost of providers with respect to their geographic area and consumer satisfaction insurance scores. This may be explained by the fact that providers with satisfied patients may increase their prices. Of course, the charged prices are not so important, as Medicare and Medicaid generally have fixed compensations per procedure.

Our results on the lack of correlation of patient reviews score and treatment quality metrics for various conditions may indicate that patients who are satisfied with their provider may not necessarily have better health outcomes, as studies have shown that patients often rate their providers based on non‒outcome-related attributes such as wait and visit times. For instance, research has shown that the average satisfaction score for wait times of 0-15 minutes was 94.3 on a 100-point scale [20].

Our findings can be used to help consumers make informed choices about their insurance plans. Health insurance marketplaces may find patient review scores for providers of each insurance plan to be a useful addition to other insurance plan metrics. Alternatively, consumers can use this information in their own research to identify potential insurance plans based on the review scores of providers on review sites such as Vitals and Healthgrades.

Further, insurers may use our results to better understand the relationship between their patients’ satisfaction and their network of providers. For example, although it is not clear if there is a cause-effect relationship, our results indicate that hiring a provider with high patient review scores may contribute more to the overall consumer satisfaction insurance plan rating than hiring a provider who has been receiving many referrals from their colleagues. Further, our results indicate that more expensive providers are correlated with higher plan satisfaction, which seems to be at odds with the providers’ “tier-ing” approach of insurers, who try to encourage patients to visit the cheaper providers.

Health care providers may also use our results to decide which insurance plans to accept. As noted above, a patient whose bill was not covered by an insurance company may complain about the billing at the provider’s office on a provider review site, leading to a lower overall patient review score. A provider wishing to maintain a favorable score may thus choose to avoid accepting insurance plans with low consumer satisfaction scores.

### Limitations

One of our biggest limitations is that not all of the data we obtained are complete. For example, a majority of the providers have zero reviews; this is likely due to the fact that only 4% of Internet users post online reviews for providers, and previous work has shown that most providers have zero reviews [21]. Similarly, a majority of the hospitals had no ranking information. A second limitation is that we sourced our data from multiple sites such as Vitals, CMS, Healthgrades, and NCQA. We then tried to map the various attributes across these sources. However, the accuracy of these data sources cannot be guaranteed. Another limitation is that referral frequency is greatly influenced by the specialty of the provider, and hence it needs to be normalized in terms of specialty in order to be used as an effective quality measure. Also, while the Castle Connolly award is prestigious and rigorously vetted, the award is biased towards providers who have more experience.

### Conclusions

Our data-driven analysis led to several interesting findings. Higher consumer satisfaction insurance scores are correlated with their providers having better patient review scores. There also seems to be a correlation between cost of medical care and insurance ratings. However, there was negligible correlation between other quantitative attributes such as number of referrals per provider, ratio of Castle Connolly award recipients, affiliated hospitals scores, and health insurance ratings. These findings may provide new insights into what attributes should be adopted by insurance marketplaces and search portals to empower patients in a patient-centered setting.

## Abbreviations

CMS: Centers for Medicare & Medicaid Services
HCPCS: Healthcare Common Procedure Coding System
HMO: Health Maintenance Organization
NCQA: National Committee for Quality Assurance
NPI: National Provider Identifier
PPO: Preferred Provider Organization
